# Linguistic features of Bipolar Disorder and Approaches: Scoping Review

**DOI:** 10.1192/j.eurpsy.2025.1062

**Published:** 2025-08-26

**Authors:** S. Lee, S. Choi, Y. Lee

**Affiliations:** 1 Psychology, Duksung Women’s University, Seoul; 2 Psychotherapy, Dankook University, Cheonan-si, Korea, Republic Of

## Abstract

**Introduction:**

A linguistic feature is a common characteristic associated with various mental disorders. In particular, bipolar disorder is one of the disorders in which verbal abnormalities as symptoms can be prominent. As technology advances and big data processing becomes easier, studies on the linguistic characteristics of bipolar disorder are increasing. However, the results of previous generations, who studied the linguistic features of bipolar disorder without computer-based methods are not considered, and have not been integrated with current research findings. It is necessary to review what methodologies can be used and what limitations should be considered to explore the linguistic characteristics of bipolar disorder.

**Objectives:**

This scoping review aims to explore how we can analyze linguistic features of bipolar disorder by using textual language. It reviewes the approaches and results of the studies can explain bipolar disorder appropriately.

**Methods:**

The study protocol follows the PRISMA-ScR (Preferred Reporting Items for Systematic Reviews and Meta-Analyses Extension for Scoping Reviews) guidelines. Research papers for this review were collected by 3 electronic databases: Google Scholar, PubMed, Web of Science Using inclusion and exclusion criteria, all investigators screened the abstracts and full texts of the articles and extracted the data independently.

**Results:**

We identified 315 potential studies from 3 databases. After screening the abstracts and full texts of articles, 17 (5.3%) articles met our inclusion criteria. We extracted elements such as results, and methodology from the above articles. The purpose of investigating linguistic features is mostly to predict and classify bipolar disorder, and to characterize the linguistic feautres of patients with bipolar disorder and other disorders. The most commonly used methodology is computer-based language analysis, such as natural language processing and learning models. Non-computer based articles, some of them adopted qualitative analysis. We confirmed the following results regarding the linguistic characteristics of bipolar disorder: a reduction in lexical variety, a higher rate of coordinate phrases, words related to power and achievement, and themes of humor and anger. In the narrative description of this, we include discussions on ethical issues.

**Conclusions:**

This scoping study synthesizes a study analyzing the linguistic characteristics of patients with bipolar disorder for the purpose of classification and discrimination. Past and present study uses various collection targets such as letters and web-based data, and adopts various methodologies including computer-based methods and qualitative analysis. In this study, we review the points to consider in order to interpret and apply the results of these studies from a clinical point of view. Additionally, the focus is presented to researchers who analyze the linguistic characteristics of bipolar disorder.

**Disclosure of Interest:**

None Declared

